# Associations with baseline visual acuity in 12,414 eyes starting treatment for neovascular AMD

**DOI:** 10.1038/s41433-022-02208-x

**Published:** 2022-08-26

**Authors:** S. D. Relton, G. C. Chi, A. J. Lotery, R. M. West, Real world AMD treatment outcomes EMR User Group, C. Santiago, H. Devonport, C. Bailey, I. Dias, P. Scanlon, L. Downey, I. Pearce, H. Saedon, S. J. Talks, B. Mushtaq, C. Brand, M. McKibbin

**Affiliations:** 1 Leeds Institute of Health Sciences, Faculty of Medicine and Health, University of Leeds, Leeds, UK; 2 Genentech, South San Francisco, CA USA; 3 University Hospital Southampton NHS Foundation Trust, Leeds, UK; 4 Leeds Teaching Hospitals NHS Trust, Leeds, UK; 5 NHS Grampian, Aberdeen, UK; 6 Bradford Teaching Hospitals NHS Foundation Trust, Bradford, UK; 7 University Hospitals Bristol NHS Foundation Trust, Bristol, UK; 8 Calderdale and Huddersfield NHS Foundation Trust, Huddersfield, UK; 9 Gloucestershire Hospitals NHS Foundation Trust, Gloucester, UK; 10 Hull University Teaching Hospitals NHS Foundation Trust, Hull, UK; 11 Liverpool University Hospital NHS Foundation Trust, Liverpool, UK; 12 Manchester University NHS Foundation Trust, Manchester, UK; 13 Newcastle Upon Tyne Hospitals NHS Foundation Trust, Newcastle upon Tyne, UK; 14 Sandwell and West Birmingham Hospitals NHS Trust, West Bromwich, UK; 15 Sheffield Teaching Hospitals NHS Foundation Trust, Sheffield, UK

**Keywords:** Prognosis, Risk factors

## Abstract

**Aims:**

To determine baseline visual acuity before the start of treatment for neovascular age-related macular degeneration (AMD), compare median and visual acuity states between treatment sites and investigate the association of socio-demographic and clinical characteristics with baseline acuity.

**Methods:**

Anonymised demographic and clinical data, collected as part of routine clinical care, were extracted from electronic medical records at treating National Health Service (NHS) Trusts. Analyses were restricted to eyes with baseline visual acuity recorded at treatment initiation. Associations with baseline acuity were investigated using multivariate linear regression.

**Results:**

Analysis included 12,414 eyes of 9116 patients at 13 NHS Trusts. Median baseline acuity was LogMAR 0.46 (interquartile range = 0.26–0.80) and 34.5% of eyes had good acuity, defined as LogMAR ≤0.3. Baseline acuity was positively associated with second-treated eye status, younger age, lower socio-economic deprivation, independent living, and female sex. There was little evidence of association between baseline acuity and distance to the nearest treatment centre, systemic or ocular co-morbidity. Despite case-mix adjustments, there was evidence of significant variation of baseline visual acuity between sites.

**Conclusions:**

Despite access to publicly funded treatment within the NHS, variation in visual acuity at the start of neovascular AMD treatment persists. Identifying the characteristics associated with poor baseline acuity, targeted health awareness campaigns, professional education, and pathway re-design may help to improve baseline acuity, the first eye gap, and visual acuity outcomes.

## Introduction

Visual acuity change and state after intra-vitreal therapy for neovascular age-related macular degeneration (NvAMD) are associated with baseline patient characteristics, the ocular phenotype, and key care processes [1–5]. However, the strongest association is with baseline visual acuity [6]. Eyes with good visual acuity at the start of treatment may have smaller gains with treatment but are more likely to retain a good visual state [5]. Early diagnosis and prompt initiation of treatment are important to maximise the likelihood of retaining or achieving a good visual acuity state [7].

Average baseline acuity in treatment-naïve eyes reported in real-world studies typically ranges from 53-57 ETDRS letters [4, 5, 8, 9]. A doubling of the visual angle is expected in untreated eyes with NvAMD in the first year after initial presentation [7]. A new diagnosis of NvAMD in the second eye is often made earlier, either during treatment of the first eye or some years later but in a patient who recognises the key symptoms and their importance and knows how to navigate the healthcare system quickly [9]. For patients with first eye involvement, the time from the onset of disease to recognition of symptoms, initial referral, diagnosis, and the start of treatment can be prolonged.

Since 2009, primary care optometrists in the UK have been encouraged to refer cases of suspected NvAMD urgently and directly to secondary care. More recent guidance from the National Institute for Healthcare and Clinical Excellence recommended referral from primary care within 24 hours of presentation and treatment in secondary care, when appropriate, within 14 days of receipt [10]. This guidance should lead to a more uniform provision of care but pooled real-world data has suggested variation in the baseline visual acuity between centres [9, 11]. Poor baseline visual acuity may be the result of delays in different stages of patient journey, including initial presentation to primary care, referral to secondary care, diagnosis, and the initiation of treatment [12, 13]. As a result, median visual acuity at presentation may be a measure of the quality of the referral pathway [9]. This study investigated variation in baseline visual acuity and clinical and socio-demographic characteristics associated with poor acuity at the start of treatment that could be addressed in targeted campaigns to raise awareness and shorten the time to diagnosis and treatment.

## Materials and methods

Anonymised socio-demographic and clinical data, collected as part of routine clinical care, were extracted from the Medisoft electronic medical record (EMR) (Medisoft Ophthalmology, Leeds, UK) at 13 National Health Service (NHS) Trusts. To be eligible for inclusion in the extraction, patients were required to have started treatment for NvAMD with anti-vascular endothelial growth factor (VEGF) therapy in one or both eyes between 1 January 2017 and 31 December 2018 and to have been 55 years or older at the time of the first injection. All eligible eyes were included: for patients with 2 eligible eyes, each eye was analysed separately but both eyes were included. (When treatment was started in both eyes on the same date, both eyes were assigned first-treated eye status in the analysis.)

Prior to data extraction, written approval from both the medical retina lead and Caldicott Guardian (responsible for data protection) at each site was obtained. Analyses of anonymised databases are classified as service evaluations by the Health Research Authority and so NHS research ethics committee approval is not required (http://www.hra-decisiontools.org.uk/research/). The project was approved by the University of Leeds Medicine and Health Faculty Research Ethics Committee (MREC 19008) and was conducted in accordance with the Declaration of Helsinki and the UK Data Protection Act.

Data extracted from the EMR for all patients included age at the start of treatment, gender, systemic co-morbidity, ocular co-morbidity, and first or second treated eye status. The presence of any co-morbidity relied on prior recording within the EMR used in secondary care. There was not a link to extract data from the primary care records, held outside of each NHS Trust. Index of multiple deprivation (IMD), an indicator of neighbourhood socioeconomic status, and the linear distance in miles between the residential address at the time of data extraction and the nearest treatment centre were calculated for each patient prior to the data extraction, using the first half of the patient postcode at the time of data extraction. Assisted or independent living status was recorded for each patient by checking their postcode against the list of care homes, with or without nursing care, regulated by the Care Quality Commission (https://www.cqc.org.uk/guidance-providers/regulations-enforcement/service-types#care-homes-nursing).

Visual acuity was recorded with habitual correction. Acuities recorded in Snellen format were converted to LogMAR but with a single decimal place. When the LogMAR acuity or ETDRS letter score was recorded using an ETDRS chart at 2 metres, these values were recorded to 2 decimal places. Visual acuities of count fingers or worse were converted to LogMAR 1.7 (ETDRS letter score of zero).

As baseline acuity was not normally distributed, the number of eyes treated in the study period was identified for each site and the median baseline LogMAR acuity was calculated. The proportion of eyes in the following categories was also recorded for each site: LogMAR ≤ 0.3 (≥70 ETDRS letters), LogMAR 0.32 to 0.58 (69 to 56 ETDRS letters), LogMAR 0.60 to 0.98 (55 to 36 ETDRS letters) and LogMAR ≥1.0 (≤35 ETDRS letters).

### Statistical analysis

Baseline characteristics of the cohort are presented by person or eye as appropriate, given that both eyes were treated and eligible for inclusion for some patients. Analyses were performed at the level of individual eyes, and so the baseline vision of each eye, if both eyes of a person were eligible, would be used separately. Univariate analysis was performed to explore variation in baseline visual acuity, recorded using LogMAR score, at each site and by first/second treated eye status. All analysis was performed using Python and R.

Association between clinical and socio-demographic variables and presenting visual acuity was further investigated using a multivariate linear regression model. Continuous variables such as age and distance from the treatment centre were first modelled with splines, using curves to model any nonlinear effects. Both were replaced with linear terms for simplicity, as a straight line would fit within the confidence intervals of the fitted splines. IMD decile was modelled as a categorical variable. The treatment site was initially modelled using random effects, though using fixed effects had little impact on the model fit and Akaike Information Criterion (AIC), so fixed effects are presented for ease of interpretation [14]. Similarly, hierarchical random effects were investigated for nesting eyes within patients but did not significantly impact the AIC and were thus removed for simplicity.

Missing data accounted for <5% of the overall dataset (primarily missing IMD and/or calculated distance from the treatment centre), and therefore complete case analysis was used. Model fit was investigated by inspecting the residuals and calibration. A global shrinkage factor was calculated and applied to the model parameters using 1000 bootstrap samples (resampling from the original data, with replacement, and rebuilding models on the samples) to improve the generalisability of the model [15].

## Results

Baseline data was available for 9406 patients (12,770 eyes) that met the eligibility criteria at the 13 NHS sites. Analysis of associations with baseline visual acuity was limited to 9116 patients (12,414 eyes) with complete baseline data. Treatment was started in both eyes on the same date in 66 patients.

The median age of the 9116 patients at the start of treatment was 81.4 years (interquartile range [IQR] = 75.3–86.4) and 5711 (62.6%) were female. Other baseline characteristics are provided in Table 1.

**Table 1:** Baseline characteristics for the 9116 patients and 12,414 eyes.

People	Characteristic	Value
Sex *n* (%)	Female	5711 (62.6%)
	Male	3405 (37.4%)
Ethnicity *n* (%)	White/British/Irish	6682 (73.3%)
	Asian/Indian/Pakistani/Mixed	59 (0.6%)
	African or Afro-Caribbean	0 (0%)
	Not recorded or unknown	2375 (26.1%)
Living status *n* (%)	Independent living	8578 (94.1%)
	Assisted living	524 (5.75%)
	Not recorded or unknown	14 (0.15%)
IMD *n* (%)	Decile 1 (most deprived)	1178 (12.9%)
	Decile 2	721 (7.9%)
	Decile 3	697 (7.6%)
	Decile 4	803 (8.8%)
	Decile 5	815 (8.9%)
	Decile 6	889 (9.8%)
	Decile 7	1011 (11.1%)
	Decile 8	937 (10.3%)
	Decile 9	888 (9.7%)
	Decile 10 (least deprived)	1152 (12.6%)
	Not available or unknown	25 (0.3%)
Median distance in miles to nearest treatment centre (IQR)		4.52 (2.68, 8.36)
	Not recorded or unknown	32 (0.4%)
**Eyes**		
Median age at baseline-years (IQR)		81.4 (75.3, 86.40)
Treated eye status *n* (%)	First treated eye	9182 (74.0%)
	Second treated eye	3232 (26.0%)
Any ocular co-morbidity present at baseline *n* (%)		3385 (27.3%)
Any systemic co-morbidity present at baseline *n* (%)		5734 (46.2%)

The total number of eyes starting treatment at each site between 2017-2018 ranged from 465 to 1,588. Median baseline visual acuity was LogMAR 0.46 (63 ETDRS letters). Visual acuity at the start of treatment was good, defined as LogMAR ≤0.3 (≥70 ETDRS letters), in 34.5% of eyes and poor, defined as LogMAR ≥1.0 (≤35 ETDRS letters), in 17.5% of eyes. Median baseline acuity for the treated eyes at each site ranged from LogMAR 0.4 to 0.5 and the proportion of eyes with good visual acuity ranged from 26.9 to 43.8% (See Table 2). Median baseline visual acuity was better for second-treated eyes, with 44.2% retaining good visual acuity state at the start of treatment, compared to 31.2% of first-treated eyes (See Table 3).

**Table 2:** Number of eyes, median, and categories of visual acuity at the start of treatment.

Site	Aggregate	A	B	C	D	E	F	G	H	I	J	K	L	M
Eyes (*n*)	12,414	633	799	465	605	1353	839	1588	1416	1209	1084	835	832	756
Median baseline LogMAR (IQR)	0.46 (0.26, 0.80)	0.50 (0.30, 0.82)	0.40 (0.20, 0.72)	0.50 (0.26, 0.90)	0.40 (0.22, 0.70)	0.48 (0.22, 0.82)	0.46 (0.30, 0.80)	0.46 (0.30, 0.80)	0.42 (0.22, 0.78)	0.50 (0.26, 0.86)	0.42 (0.28, 0.78)	0.50 (0.30, 1.00)	0.50 (0.30, 0.90)	0.48 (0.30, 0.80)
LogMAR ≤ 0.3 (%)	34.5	26.9	43.8	36.1	38.7	34.6	32.9	35.0	38.4	32.3	32.7	32.8	35.0	27.9
LogMAR 0.32 to 0.58 (%)	25.0	28.0	21.5	17.4	26.0	24.3	26.9	26.6	24.8	24.5	28.7	19.2	21.2	32.0
LogMAR 0.60 to 0.98 (%)	23.0	26.5	22.0	24.5	22.8	24.4	23.6	21.2	21.7	23.9	24.3	20.0	22.7	23.7
LogMAR ≥ 1.0 (%)	17.5	18.6	12.6	21.9	12.6	16.7	16.6	17.2	15.1	19.3	14.4	28.0	21.2	16.4
First eyes ***n*** **(%)**	74.0	72.5	74.3	76.1	75.0	74.7	69.0	74.4	73.2	75.1	72.1	75.9	77.0	72.5

*IQR* Inter-quartile range.

**Table 3:** Number of first and second treated eyes, median, and categories of visual acuity at the start of treatment.

	First eyes	Second eyes
**Eyes (*n*)**	9182	3232
Median baseline LogMAR (IQR)	0.50 (0.30, 0.88)	0.38 (0.20, 0.64)
LogMAR ≤ 0.3 (%)	31.2	44.2
LogMAR 0.32 to 0.58 (%)	24.6	26.1
LogMAR 0.60 to 0.98 (%)	24.3	19.2
LogMAR ≥ 1.0 (%)	19.9	10.6

**Fig. 1:**
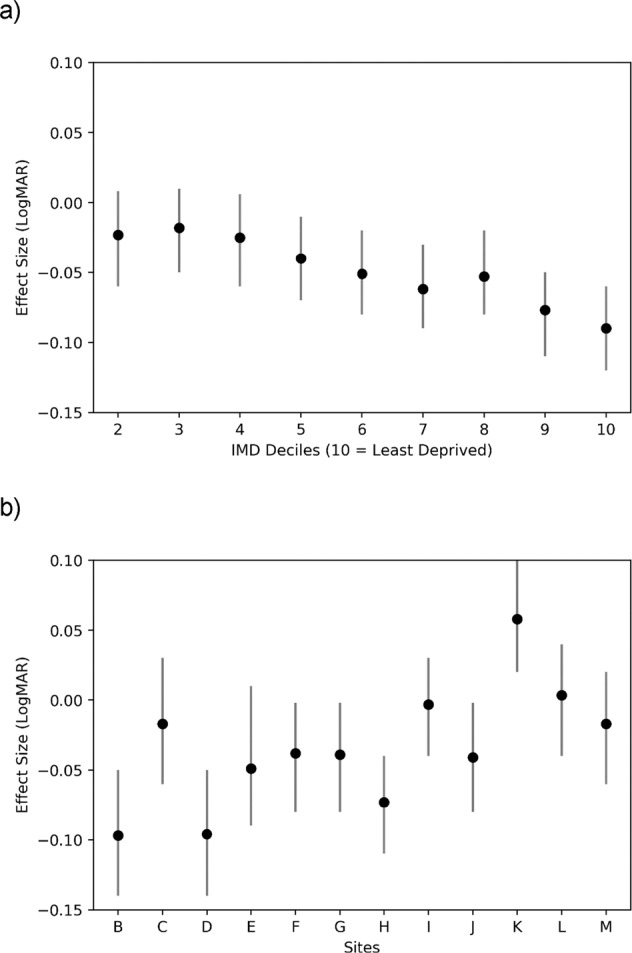
Effect size of both Index of Multiple Deprivation (IMD) and Site on baseline LogMAR visual acuity in multivariate regression analysis. The effect of IMD is shown in (**a**) and of treatment site in (**b**), with error bars to show the 95% confidence intervals.

The results of the multivariate linear regression are shown in Table 4. The effects shown are those found after shrinkage by the global shrinkage factor of 0.957, indicative of a good initial model fit. Compared to second eyes, baseline acuity in first treated eyes was worse by LogMAR 0.15 (95% CI: 0.13 to 0.16) or 7.5 ETDRS letters. Compared to the eyes of people living in the most deprived areas (IMD 1), baseline acuity in the eyes of people living in the least deprived areas (IMD 10) was better by LogMAR 0.09 (95% CI: 0.12 to 0.06) or 4.5 ETDRS letters (See Fig. 1a). A similar increase in baseline acuity was associated with independent living status and for every 10-year reduction in age at the start of treatment. (Table 4) Compared to site A, median baseline visual acuity was also better by almost LogMAR 0.1 (5 ETDRS letters) at sites B and D (See Fig. 1b). There was little evidence of association between baseline acuity and distance to the nearest treatment centre, systemic or ocular co-morbidity.

**Table 4:** Regression analysis showing associations of clinical and socio-demographic characteristics with baseline visual acuity.

Parameter		Estimate	95% CI	*p*-value
Intercept		−0.1414	(−0.23, −0.05)	
Age (decade)		0.094	(0.08, 0.1)	<0.001
Male sex (vs Female sex)		0.015	(0.001, 0.03)	0.04
Independent living status (vs Assisted)		−0.09	(−0.12, −0.06)	<0.001
Distance from treatment centre		0	(−0.0005, 0.0004)	0.87
First eye (vs second eye)		0.15	(0.13, 0.16)	<0.001
Ocular comorbidity (present versus absent)		−0.004	(−0.02, 0.01)	0.65
Systemic comorbidity (present versus absent)		0.0033	(−0.01, 0.02)	0.68
Site (relative to A)				
	Site B	−0.097	(−0.14, −0.05)	<0.001
	Site C	−0.017	(−0.06, 0.03)	0.47
	Site D	−0.096	(−0.14, −0.05)	<0.001
	Site E	−0.049	(−0.09, 0.01)	0.011
	Site F	−0.038	(−0.08, −0.002)	0.066
	Site G	−0.039	(−0.08, −0.002)	0.038
	Site H	−0.073	(−0.11, −0.04)	<0.001
	Site I	−0.0033	(−0.04, 0.03)	0.867
	Site J	−0.041	(−0.08, −0.002)	0.04
	Site K	0.058	(0.02, 0.1)	0.006
	Site L	0.0033	(−0.04, 0.04)	0.88
	Site M	−0.017	(−0.06, 0.02)	0.42
IMD (deciles relative to 1 (most deprived))				
	IMD 2	−0.023	(−0.06, 0.008)	0.14
	IMD 3	−0.018	(−0.05, 0.01)	0.28
	IMD 4	−0.025	(−0.06, 0.006)	0.11
	IMD 5	−0.04	(−0.07, −0.01)	0.01
	IMD 6	−0.051	(−0.08, −0.02)	<0.001
	IMD 7	−0.062	(−0.09, −0.03)	<0.001
	IMD 8	−0.053	(−0.08, −0.02)	<0.001
	IMD 9	−0.077	(−0.11, −0.05)	<0.001
	IMD 10	−0.09	(−0.12, −0.06)	<0.001

## Discussion

This study confirms ongoing variation in both median visual acuity and the percentage of eyes with good visual acuity state at the time that NvAMD treatment was started at 13 NHS treatment sites. Moreover, baseline visual acuity varied between sites in linear models despite case-mix adjustment for covariates such as patient demographics, IMD, and independent living status.

Despite the variation between sites, a positive finding is that median baseline visual acuity for eyes treated within the publicly funded NHS may be improving. For 9243 eyes starting treatment in 2012 and 2013, median baseline acuity was 0.60 LogMAR and Talks *et al* reported a median baseline acuity of 0.56 LogMAR for 5,630 eyes starting treatment between 2013 and 2015 [9, 11]. Evidence of better visual acuity outcomes for eyes starting treatment with good baseline acuity and updated NICE guidance may have contributed to this trend [10]. Compared to 17.5% of eyes starting treatment between 2013 and 2015, almost 35% of the eyes in this data extract had a good baseline visual acuity [9].

As reported by others, median baseline visual acuity was better for second-treated eyes than for first-treated eyes [9, 16, 17]. The size of the “first eye gap” reported here is broadly similar to the mean difference reported by others [16, 17]. New disease in the second eye is often identified with structural OCT imaging or clinical examination before symptoms develop, especially for extra-foveal disease [18]. Tests of self-reported changes in vision are less sensitive than OCT imaging in identifying second eye disease [19]. Detection of second eye disease during treatment for the first eye is associated with better baseline acuity than when detection is delayed until after first eye treatment has been concluded [17]. Better baseline acuity in the second eye may result in smaller acuity gains with treatment but is key to ensure that these eyes retain a good visual acuity state [18].

Reducing the “first eye gap” in terms of presenting visual acuity is a key element of improving visual acuity outcomes. A recent national survey of patients with a new diagnosis of AMD found that fewer a third of patients sought help after developing symptoms [13]. Most of those with symptoms, especially women, sought help quickly but delays can be seen with men, the elderly, and those without private health insurance [13, 20]. Parfitt’s national survey found that the majority of newly diagnosed cases were found to have visual impairment or other signs of AMD at a routine visit to an optometrist [13]. Regular access to structural OCT imaging in the community for the elderly may help to identify new and often pre-symptomatic NvAMD.

Other key independent associations with baseline median visual acuity were age, socio-economic deprivation, and independent living status. For every extra 10 years of increasing age at the start of treatment, the median baseline visual acuity was worse by almost 0.1 LogMAR (5 ETDRS letters). Similarly, residence in assisted living accommodation at the time of data extraction was also associated with worse median baseline visual acuity of almost 0.1 LogMAR. The same difference was noted for the eyes of people living in the areas of highest socio-economic deprivation (1st IMD decile), when compared to those living in the least deprived areas (10th IMD decile). More *et al* have also reported an association between increasing deprivation and presentation with severe visual impairment [21].

At presentation, median visual acuity differed by 0.1 LogMAR (5 ETDRS letters) between the 13 sites and there was almost a 17% difference in the proportion of eyes with good acuity at the start of treatment. While some of this difference could be explained by other characteristics, regression analysis found that the variation between sites remained even after case-mix adjustment. Median baseline acuity was better at sites B and D by almost 0.1 LogMAR when compared to sites A and K. The reasons for this variation are not clear but individual and professional awareness, local access to healthcare systems, the efficiency of both the referral process and the pathway to start treatment, and local commissioning policy may all be implicated. The Medisoft EMR does not allow capture of the date of referral and the extraction did not allow identification and comparison of variation or delays between diagnosis and the start of treatment. Prior to the 2018 update, NICE guidance had been to limit treatment to eyes with baseline acuity between Snellen 6/12 and 6/96, although real-world evidence suggests that local commissioning policies may have been different, both before and during the inclusion period for this study [10, 22].

Targeted health awareness campaigns to stress the importance of new distortion and deteriorating vision may help to reduce the time to initial presentation [12]. Similarly, education of staff in assisted living accommodation and healthcare professionals in primary care is required to ensure prompt initial assessment and onward, direct referral for diagnosis and treatment [12]. The data from this study suggest that these measures are needed most in areas with low median baseline acuity, with a high proportion of elderly residents and high levels of socio-economic deprivation. Organisations providing treatment may also need to ensure adequate capacity to assess patients referred with suspected wet AMD and to initiate treatment, when appropriate, promptly [12]. A switch to contacting patients by phone or email may be required to reduce delays reported with more traditional communication by post [13]. Routine follow-up of patients treated in the first eye should include measurement of visual acuity and structural OCT imaging of the fellow eye [10]. Patients should be instructed verbally and given a printed reminder to seek help in the event of new symptoms in the second eye, after treatment has been paused or stopped in the first eye. Adequate capacity and improved patient communication should become a requirement of commissioning policy [10, 22].

The use of pooled data from many patients at multiple sites with wide geographical coverage adds validity to the findings of this study and improves the generalizability of study findings to the underlying UK NvAMD population. Several of the key findings are also supported by other publications. A potential weakness is that the postcode used to determine IMD, distance to the nearest treatment centre, and assisted living status was that at the time of data extraction and not necessarily at the start of treatment. A single postcode covers an average of 15 properties but the total can be up to 100 in areas of high-density accommodation. IMD ranking is produced for small areas, using seven domains of deprivation. These areas are designed to be of similar population size, with an average of 1500 residents or 650 households. Therefore, data derived from postcodes and IMD areas is an average of all the properties at that location and may not be fully applicable to each individual household. In addition, 26% of the eyes were second-treated eyes and so the effect of the socio-economic and living status variable would be repeated in the eye-level modelling. To control for the potential bias that this doubling would introduce, a sensitivity analysis using a random effect for each patient was performed and found to make almost no difference. As the data extracted for this study were recorded as part of routine clinical practice in the publicly funded NHS in the UK and so may not be applicable to other healthcare systems. Other factors that may be associated with lower baseline VA, such as patient recognition of symptoms, referral speed, and capacity at the treating centre, particularly in relation to minimising delays between diagnosis and the start of treatment, were not available in the EMR and could not be analysed in this study.

Although median baseline visual acuity among NHS patients may be improving, this study provides evidence of ongoing variation in median acuity and visual acuity state between NHS treatment centres at the start of NvAMD treatment. Baseline visual acuity was positively associated with second-treated eye status, younger age, lower socio-economic deprivation, and independent living. Targeted health awareness campaigns and faster access to diagnosis and treatment may help improve baseline acuity, reduce the first eye gap and enhance treatment outcomes.

### Summary Table

#### What was known before

- Visual acuity outcomes after treatment for neovascular AMD are influenced by baseline visual acuity.
- Baseline visual acuity is associated with age, deprivation, sex, and first or second treated eye status.

#### What this study adds

- Baseline visual acuity in the publicly funded NHS may be improving.
- The gap in median acuity at presentation between first and second treated eye is 6 EDTRS letters.
- Even with adjustment for other variables, variation in baseline visual acuity between treatment centres persists.

## Data Availability

Sharing or independent access to the data analysed here is not possible as Caldicott guardian approval at each participating site was limited to the data controller (MM) and staff at the Leeds Institute of Health Sciences (SDR and RMW).
